# Supplementary material for: Variational-LSTM autoencoder to forecast the spread of coronavirus across the globe
Source: PLoS One. 2021 Jan 28;16(1):e0246120. doi: 10.1371/journal.pone.0246120 (PMC7842932; doi:10.1371/journal.pone.0246120)
Supplement: Attachment — Submitted filename: Response to reviewers.docx [file pone.0246120.s002.docx]

Dear Editor and reviewers,

Thanks for your comments and feedback. We have modified several aspects in the article:

1- We have updated the data to June, to make the article up-to-date.
2- We have applied a logarithmic scale for the outputted data alongside the actual scale (linear scale). We believe that both have very different applications. Understanding the pattern is one thing while giving the ability to predict actual values is also vital for many applications.

On a side note, a logarithmic function tends to smooth out any given function, by reducing the data dimensionality and removing outliners. This makes it simpler to forecast than the actual number, unlike what reviewer 2 mentioned (In fact, a log output would tend to show a better performance of our model than the actual data). Please, see the added figures. The log functions tend to widen the difference of the earlier points of low values, whereas close the different at the end of the curve. This shows how the model learns the pattern of the data after the initial zero cases of most countries (after having enough cases to extract patterns).

3- Besides our evaluation metrics, we have added also a Mean squared logarithmic error (MSLE) to compare the results of different countries. It is a relative error, which could be a good indicator for comparing the performance of the model in different countries despite how large or small the values of the cases in a country.
4- We have added two new discussion points: to discuss outliners, and the online inference of the model and its flexibility to different data sources as suggested by reviewers 1

All figures including maps are within our copyrights, we did not use google maps or any copyrighted software to visualise them. We created them using open-access python libraries- Basemap and Matplotlib.

Please, see a detailed response to each comment below.

Additional Editor Comments:

The paper shows a model that is interesting (see comment of reviewer 1). However, the manuscript makes extravagant claims. It also misleads by using linear scale whose results in the most part appear as being close with the real data while in reality are far in a log scale (see comments of reviewer 2). I recommend to better apply log scales and to claim more realistic comments closer to the data, emphasizing the limits of the model

We have applied a logarithmic scale for the outputted data alongside the actual scale (linear scale). We believe that both have very different applications. Understanding the pattern is one thing while giving the ability to predict actual values is also vital for many other applications that require a short-term forecast of accurate estimation per day.

We also discussed more points to emphasise the limits of the model in the discussion section.

**Reviewer #1:**

The study reports a model that predicts trends of the spread of coronavirus at a country level which will be of immense utility value for policymakers and public health planners.
An added utility is that it is based on machine learning and will complement other mathematical and statistical models.
The novelty of the model is that it includes historical data, location, demographic data, and more importantly government measures to mitigate the epidemic (e.g. cancelled public events, travel restrictions, closing of work-places, closure of international travel etc.) and spatial dependence of states to each other. This improves the validity of the model to higher levels of precision. The use of country-specific data has enhanced its relevance and applicability to policymakers at a country level. Its flexibility and ability to refine to the city level is a distinct advantage, because the epidemic often begins in localities and spreads within cities.
The paper is well written and easy to understand. The tables and figures are excellent. The references are up-to-date.

Two points could be explored. How would the model respond to further data on the effectiveness of measures (e.g. travel restrictions as a measure vs. real-time mobility data as an outcome of the measure)?

This is an interesting and valid point for discussion. The model is trained on data from different countries, with different measures applied at a different pace. It has also seen data before, during and after lockdown measures of different regions. Moreover, the model has seen data before travel restriction and after travel restriction (from January to June) for certain countries. Accordingly, even if measures and restrictions change in future, this could allow the model to predict future cases by understanding and inferring the change in the measures and their effects in a given country. However, as for the future improvement of the model, more data types -when they are available- could be feed for the model. Besides the policies and restriction that would vary from country to country, real-time mobility data or mobility indices could help the model to forecast new cases in the post-lockdown periods.

Building on how the model could be used for future inference, deploying the model in an online platform is a possible application of this research. This application could assist policy-makers to have a better overview picture of the status of the spread of the virus across the globe, nevertheless, implement or eliminate a given policy. In table 3, we show the run-time required for the different tasks on a single GPU. We show that updating the data, computing adjacency, fusing the data, and re-training the model would require less 5 min, whereas utilising the model in the inference model will require less a second to predict a future step (multi-steps) for all countries.

We have added a discussion point in the discussion section, addressing how the model can be used for online inference in a data stream.

How well would the model fit with countries that are outliers (e.g. Sri Lanka has had less than 1350 cases as of 26th May and 10 deaths. This includes 650+ cases detected within a navy camp and about 300 in Sri Lankan returnees who were working overseas, especially from the Middle East. This demonstrating that their strict policy of curfews coupled with detect, trace contacts, quarantine have essentially limited the spread to clusters with almost zero community spread (https://epid.gov.lk/web/).

This seems to be an interesting case. We have discussed the limitation of the model to outliers, in the discussion section. We also added the case of Sri Lanka in the results of the single-step and multi-step models to show the performance of the model and how the model performs in countries with fewer cases.

Overall, the model would perform less accurately in countries with fewer data points for a given country (countries with a spread that started at a very late stage). However, the model would not be affected by how high or low the values per day as long as there are enough data points for a country to learn its pattern.

Based on our experiments, the model still understands the pattern in countries that are perceived as outliers, but with lower accuracy. For example in case of Sri Lanka, the strength of the model performance, in term of r-squared, decreases by 23% percent or the logarithmic error increases by 2.5 folds in comparison to the model performance in predicting spread cases in the United States (See table 1). We further discussed this issue in 5.3 Limitation and future work

**Reviewer #2:**

The results are projected in linear figures, while it is obvious that the logarithmic scale is the one which is of interest. Where the results to be transformed in a logarithmic scale (which is of interest for projecting the cases in the next few days) the results from the authors would in many countries show differences from real data. For example, the prediction for Italy would show, in a logarithmic figure that the cases would flatten out soon, while the real data (and the ensuing history which is now known) would have shown the opposite. Countries like Russia, France, the UK and more from the ones mentioned in here, have the same (I would say almost systematic) type of behavior. That is, the model shows almost all of them with a significantly lower estimate of expected cases. Though the actual difference is small, the trend is significantly different. And the trend is more important than the actual number as it is what allows (or enforces) additional measures to be lifted (or taken).

Given that understanding the pattern is important as well as the true values, we have plotted the results in true figures and logarithmic scale. It is up to the readers- or policy-makers -to apply the model in whichever form that suit the application.

Concerning the trend more important than the actual numbers: Understanding the trend is one application of a predictive model and could be important for drawing policies or remove certain regulation, which we have provided in the new figures for each country which the model shows a good understanding of the pattern. Since that log function tends to reduce the dimensionality of data, many countries will follow the same trend, even if they have an extremely different count of cases. This also can be seen as misleading information. For instance, if you trying to estimate how many bed or ventilations in the hospitals that are needed in the next days, then the actual number per day is more vital than the pattern. Even if the log scale is transformable to a normal scale, the small loss at a log scale- if the model is trained in it- would lead to a large loss in normal scale. Nevertheless, the latter (Estimating numbers in normal scale) is missing in the literature as most of the model would rather project a pattern, than provide a predictive model that able to predict day by day.

Additionally, the manuscript makes extravagant claims such as the possibility for a 1-3 month prediction by using this method. I feel that the forecasts made in the manuscript are not as good to support such a claim.

We mentioned in the early manuscript in future work section: *“Put all together, more data, more factors, different forecasting models could also lead to better long-term forecast (1-3 months) for each country based on the lesson learned from the global and country-level trends of spread.”*

We have re-stated our statements to make sure that the scope and the limitation are clear, and how this could be reached in the future using our model.

Concerning the statement: “I feel the forecasts made in the manuscript are not as good”, we have provided several empirical methods to quantify the losses and the accuracies of our model, in the addition to the descriptive analysis of the plotted figures and maps which shows with more data in the future- similar to any machine learning models- the model would be capable of forecasting the long term pattern if fed with more data on the upcoming months, which supports our statement of the long-term forecasts.

On a separate issue, I feel that the reality has taught us that only the initial few cases of a specific country depend on how other countries are going.

We are not sure if we fully understood this statement. Initial cases could be a good indicator of how a given country can lead to- for understanding other countries. However, what happens when a country that has similar initial cases to another country, took different measures? We don’t think initial cases is enough to predict the complexity of reality as we mention in the articles each country has unique factors, measures, spread patterns, and adjacency link to other countries. Only, the combinations of this, or even more factors, could lead to better prediction.

We have clarified that in the new submission.

Upon quarantine measures are taken into effect the evolution of cases in any country depends on the strictness and enforcement of the measures taken.

We agree that strictness of the measure during the quarantine time could be an indicator however, it is never be quantified or supported by empirical methods. Nevertheless, there are other factors, even with strict enforcement that could lead to the evolution of cases in a given country. For instance, the effect of going to grocery stores is not yet measured, whereas it takes place in every country despite the enforcement of the quarantine measures and could lead to exposures. Second, the number of testing per day -despite how strict the enforcement of measures could lead to adding more numbers of cases in a given country, despite how strict the measure is. Third, people moving by car or bus to adjust countries, which we think it is the case in the EU countries, despite how strict the measures countries are taking.

Based on our empirical findings, understanding adjacency of countries has improved the prediction of the model – in comparison to without the adjacency of countries- and we have mentioned that in our method.

Countries like South Korea and the USA have had (and still have) a totally different history and under no circumstances does the case of one affect the other.

We agree that countries have different patterns due to their demographics features, urban features, spread historical data, and applied policies and measures. We never mentioned anything regarding South Korea and the USA having a similar history. However, one case elsewhere is enough to cause spread in another country by mobility despite the different nature of each county –the reality has taught us that. Understanding the adjacency between countries means including factors due to mobility that is not necessarily based on travel by flights. It could, for instance, travel by car (which could be the case across Europe, or across the US states), even during the US taking an international strict measures, but moving around the country or adjacent countries was not yet limited. Even if cases are few, they remain as important risk factors that could be neglected.
