# Supplementary material for: Variational-LSTM autoencoder to forecast the spread of coronavirus across the globe
Source: PLoS One. 2021 Jan 28;16(1):e0246120. doi: 10.1371/journal.pone.0246120 (PMC7842932; doi:10.1371/journal.pone.0246120)
Supplement: Attachment — Submitted filename: Response to reviewers.docx [file pone.0246120.s003.docx]

Dear Editor and reviewers,

Thanks for your comments and feedback. We have addressed the two comments raised by the reviewers.

*Reviewer 1: asks to mention other possible variables (maybe at the end of the paper in conclusion to explain why the model fitted lower with some cases and give future possible improvements)*

We have added discussed the variables that we found insignificant in section 3.1.2 Urban features data.

*Reviewer 2: be more modest on the result and improve the Log graphs by putting "real values": Also I would add that log graphs must have the background of lines with uneven distances, which shows  obviously at the first glance that it is log (do you see what I mean? If it is not clear I can send you an example of these kinds of graphs by email: please contact me directly)*

We have modified the interpretation of the result to be more modest by removing unnecessary words. We have changed all the logarithmic graphs to be with an [unevenly distributed y-axis](https://stackoverflow.com/questions/30234921/python-plot-unevenly-distributed-axis).

Regards,

The authors.
