# Supplementary material for: Variational-LSTM autoencoder to forecast the spread of coronavirus across the globe
Source: PLoS One. 2021 Jan 28;16(1):e0246120. doi: 10.1371/journal.pone.0246120 (PMC7842932; doi:10.1371/journal.pone.0246120)
Supplement: Attachment — Submitted filename: responses_to_reviewers_Rev3.docx [file pone.0246120.s004.docx]

Dear editor and reviewers,

We would like to thank you for your time and effort to review this article for the third round.

We added a new table (Table 1) to explore the impact of the adjacency matrices and variational autoencoder components on the global results of the model as suggested by Reviewer 3.

Reviewer 3 has suggested different discussion points with reference to several articles that have been published in July or even last month (October), whereas this presented article was submitted to the journal in April. Given the length of time our article has been under review and the unprecedented pace of progress in this field, we believe it is unfair to require us to substantially modify the paper to account for new literature at the third review round.

The article has been through three different minor revisions since the first submission and all the raised comments were minor comments from different reviewers that focus on addressing viewpoints and discussion. While we believe all of the raised comments are valuable and valid discussion points, they do not contribute to the advancement of the introduced method which is our main contribution in this article.

Please find our detailed responses and, where we feel it is necessary, rebuttals for the raised discussion points below.  We would appreciate it if the journal makes a final decision concerning the presented article.

Regards,

Authors

Reviewer #3: The paper presents a model to predict coronavirus spread based on machine learning and it could be another important tool in the hands of policymakers to better attempt to control the epidemic spread.
The model uses historical data, location, demographic data to feed the model and more importantly it uses government measures to control the epidemic and interdependency of the countries/cities to address the influence of each in the state of the epidemic of the others.
The paper is well written and is easy to understand and to the best of my knowledge is a good contribution to the COVID research landscape.
However, I think some points should be more highly discussed.

The authors claim that the use of demographic data is one of the advantages of the proposed method. In section 3.1.2 the authors present the factors from the demographic data they included in the model and how it is correlated with the covid cases spread over time, but afterwards no result is presented confirming the impact of these factors in the prediction. I have the intuition that some if not all of these factors of demographic data are somehow present in the COVID-19 confirmed cases data.

It has been mentioned in the article (Section 3.1.2) that there are two main reasons for selecting these factors: 1) their correlation with the spread, and 2) their enhancement of the model outcomes based on trial and error. The impact of the factors has been seen during the creation of the model and how the model becomes more reliable. In other words, these factors provide a unique set of features for the different countries that the model self-learn from it through the introduced algorithm. This also has been mentioned in Model Hypothesis, see section 2.1 Hypothesis and assumptions.

Following the same line of thinking, the interaction between countries, that I think is one of the most important considerations of the paper, does not explore its impact on the results of the model. I think some comparative results may be included considering different adjacency matrices, like the ones presented in section 3.1.4, and the absence of it.

We have added a new table to compare the results with and without the introduced adjacency matrix at a global scale. The results of the introduced method with the variational autoencoder component and the introduced adjacency matrix reduces the losses of the same model without these components with an RMSE loss value of 174.3 (See table 1). Also, for further analysis, the below figure shows the outcomes of the model without adjacency similar to the one introduced in the article with adjacency matrix and VAE (Figure 9). It also confirms how the results improved by applying the adjacency matrix.

Also regarding interaction between countries and adjacency matrices, in some early papers of COVID-19, for instance, the ones presented by Vittoria Colizza on the risk of importation ([https://www.thelancet.com/journals/lancet/article/PIIS0140-6736(20)30411-6/fulltext](https://eur01.safelinks.protection.outlook.com/?url=https%3A%2F%2Fwww.thelancet.com%2Fjournals%2Flancet%2Farticle%2FPIIS0140-6736(20)30411-6%2Ffulltext&data=04%7C01%7C%7C25257eef7af54cb841c408d892214a0a%7C1faf88fea9984c5b93c9210a11d9a5c2%7C0%7C0%7C637420018956357714%7CUnknown%7CTWFpbGZsb3d8eyJWIjoiMC4wLjAwMDAiLCJQIjoiV2luMzIiLCJBTiI6Ik1haWwiLCJXVCI6Mn0%3D%7C1000&sdata=vaYlVGYyhFWbpu8Qlco%2F%2BhOPVe3XIhH40kiRRjTjfUk%3D&reserved=0), [https://journals.plos.org/plosmedicine/article?id=10.1371/journal.pmed.1003193](https://eur01.safelinks.protection.outlook.com/?url=https%3A%2F%2Fjournals.plos.org%2Fplosmedicine%2Farticle%3Fid%3D10.1371%2Fjournal.pmed.1003193&data=04%7C01%7C%7C25257eef7af54cb841c408d892214a0a%7C1faf88fea9984c5b93c9210a11d9a5c2%7C0%7C0%7C637420018956357714%7CUnknown%7CTWFpbGZsb3d8eyJWIjoiMC4wLjAwMDAiLCJQIjoiV2luMzIiLCJBTiI6Ik1haWwiLCJXVCI6Mn0%3D%7C1000&sdata=XK7EDQc1Dd2UNF5kSDDSC%2BrhPFsseFf8dIdwhiophnw%3D&reserved=0)) is used the data of air travel as a proxy of the interaction. Why is this not considered in this model?

At the time of writing the article, it was not possible to obtain open access flight data at the global level in a timely fashion. Moreover, there are many more transportation modes that may be used for international travel at the continental level that would not be captured in the flight data. Attempting to collect this data on the global scale would sacrifice the timeliness of our research in a rapidly evolving situation. Therefore, we developed our method to infer mobility from the data. The purpose of using a variational autoencoder in this model along with the adjacency matrix (as explained in the paper) is to generate different weights between the countries that represent the mobility flow, not only air travel but all types of mobility. The data taught us that even when there were air travel restrictions, cases remain increasing in certain countries, for instance, among Western European countries, given that distances are shorter and people can travel with cars and coaches. Cargo flights were still active, etc. Accordingly, using air travel alone as a proxy could be a limitation as well as an advantage.

It worth mentioning that this article has been submitted to the journal in April, whereas the second article you refer to was published on July 17, 2020. Given the rapid pace of developments in this field, we feel it is a little unfair to be asked to consider new literature at the third round of review that were not mentioned in previous rounds.

Another important advantage of the model is the inclusion of governmental mitigation measures. In the paper ([https://www.thelancet.com/journals/laninf/article/PIIS1473-3099(20)30785-4/fulltext?fbclid=IwAR3tcQgOb7CUa3bAxgOL7bsqRPj5jJn-UZI4KcpzTBfIEgt98YWucuZU2Zg)](https://eur01.safelinks.protection.outlook.com/?url=https%3A%2F%2Fwww.thelancet.com%2Fjournals%2Flaninf%2Farticle%2FPIIS1473-3099(20)30785-4%2Ffulltext%3Ffbclid%3DIwAR3tcQgOb7CUa3bAxgOL7bsqRPj5jJn-UZI4KcpzTBfIEgt98YWucuZU2Zg)&data=04%7C01%7C%7C25257eef7af54cb841c408d892214a0a%7C1faf88fea9984c5b93c9210a11d9a5c2%7C0%7C0%7C637420018956367706%7CUnknown%7CTWFpbGZsb3d8eyJWIjoiMC4wLjAwMDAiLCJQIjoiV2luMzIiLCJBTiI6Ik1haWwiLCJXVCI6Mn0%3D%7C1000&sdata=qfwv7n19JUbptahhIG3l88OfGHQwWlrp55z6Jo4%2BXMQ%3D&reserved=0) it is shown that most measures have an impact on the reproduction number, and therefore in the cases after 7-14 days.

Again, the suggested article is published on October 22, 2020, whereas our paper has been submitted to the journal in April. The findings of this research were not available when the article was submitted or after previous rounds of review, nor affect the introduced method presented in this article.

Considering that the predictions of the model are somehow validated to be good till 10 days in advance there is any result that supports that the prediction is being influenced by the measures?. Could it quantify the change in the behaviour of the prediction after a governmental measure is taken?.

We think this is a good idea which we have discussed in future works. The introduced method focuses on predicting the spread cases globally, as a novel method to predict spread cases bearing in mind urban factors, governmental policies, and the adjacencies among different countries with deep learning. Quantifying the change in the behaviour and measuring the effect of governmental policies is a field of research in its own right, which it beyond the scope of the presented article (See section 5.3 Limitations and future work).
